# A Unique Case of Cutaneous Larva Migrans and Asymptomatic Löeffler’s Syndrome

**DOI:** 10.7759/cureus.15960

**Published:** 2021-06-27

**Authors:** Jason Ng, Daniel Lee, Marek Kryzstofiak

**Affiliations:** 1 Internal Medicine, Regional Medical Center Bayonet Point, Hudson, USA

**Keywords:** cutaneous larva migrans, hyper-eosinophilia syndrome, pulmonary disease, infectious and parasitic diseases, parasitic diagnosis, tropical medicine, löeffler’s syndrome

## Abstract

Cutaneous larva migrans is a fairly common parasitic skin disease typically found in more tropical climates such as the southeastern United States, with the most commonly encountered organism known as the hookworm, *Ancylostoma braziliense*. Löeffler’s syndrome is a rare pulmonary manifestation with vague diagnostic criteria but frequently characterized by diffuse migratory pulmonary infiltrates on imaging studies with accompanying serum eosinophilia. Here, we present a unique case of Löeffler’s syndrome secondary to cutaneous larva migrans.

## Introduction

Löeffler’s syndrome is a rather rare pulmonary manifestation with vague diagnostic criteria. Upon literature review [1-4], it is frequently characterized as multiple diffuse, migratory pulmonary infiltrates with concomitant serum eosinophilia. Cutaneous larva migrans, on the other hand, is a fairly common tropical parasitic skin disease [2]. In one particular study, 6.7% of travelers visiting a travel-related clinic presented with this disease [5]. It is typically found in warmer climates where the parasite seeks a host via dermal penetration. Diagnosis is frequently made clinically with careful consideration of both a patient's history and presentation [4]. This case report illustrates the rare pulmonary manifestation in a presentation of cutaneous larva migrans.

## Case presentation

**Figure 1:**
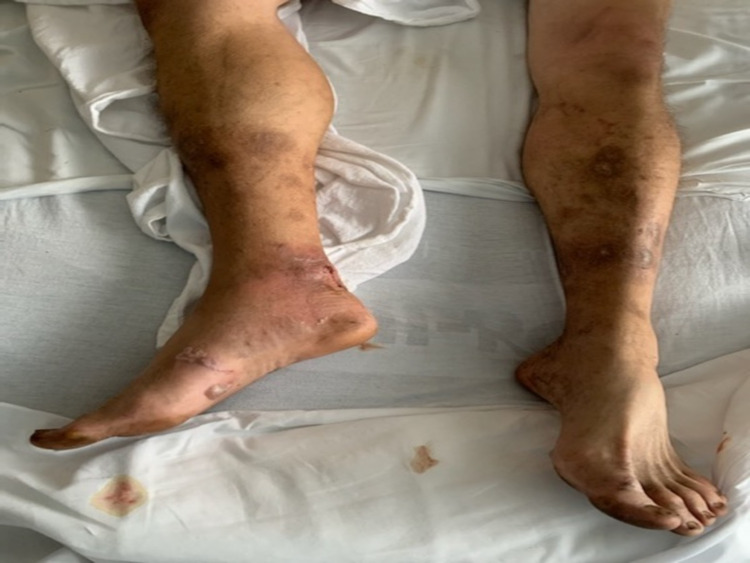
Serpiginous rash and bullae over the right foot

**Figure 2:**
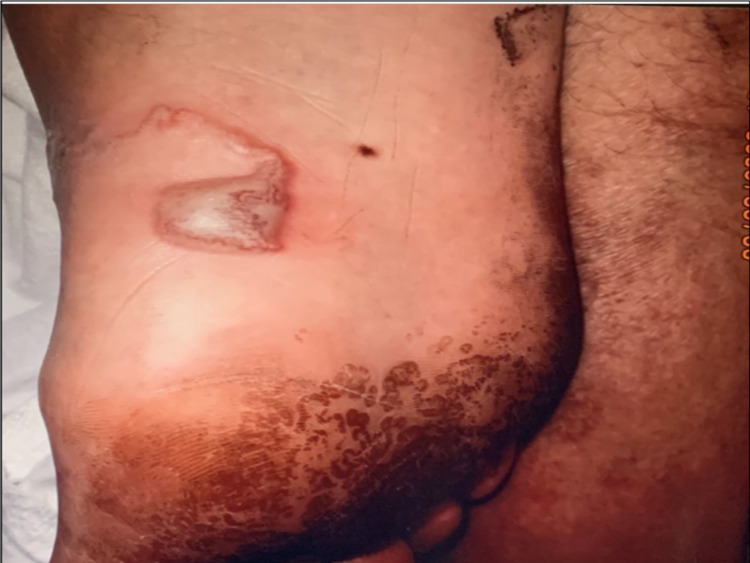
Serpiginous lesion with bullae over the right foot

**Figure 3:**
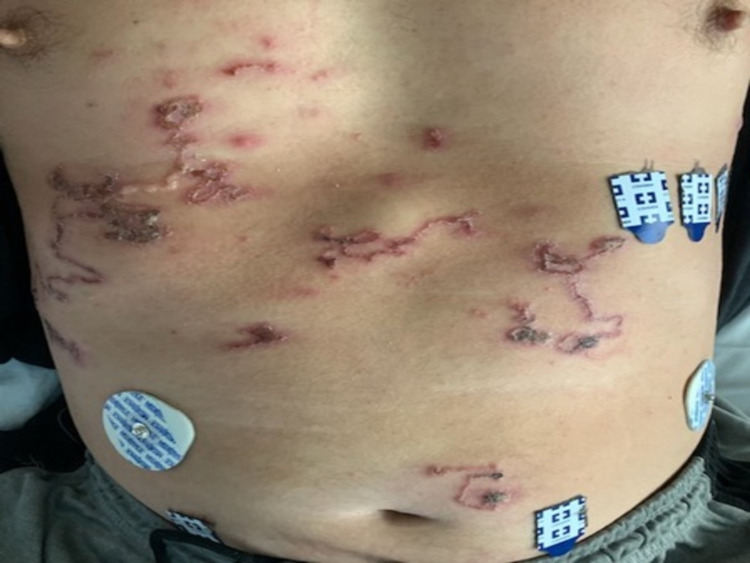
Multiple serpiginous lesions with overlying excoriations over the chest and abdomen

A 52-year-old Caucasian male presented to our hospital with complaints of ankle pain. He had a serpentine rash over his ankle which had been progressively worsening for the last several weeks with now, diffuse pruritus across his abdomen and chest, which he had been scratching for the aforementioned time period. He denied any insect bites or recent travel outside of the state of Florida but confessed to working outside - barefoot in an area where feral cats frequently defecate. The patient did not report any past medical history but did admit to a prior social history including both prior tobacco (30 pack year) and intravenous drug abuse (cocaine and amphetamine). The patient otherwise denied any active recreational drug use or alcohol intake. Upon arrival at the hospital, the patient's vital signs were temperature of 36.9°C, blood pressure of 121/70 mm Hg, pulse of 83 beats per minute, and oxygen saturation of 99% on room air. Multiple areas of serpentine tracks with erythema were noted on physical examination (Figures 1-3). 

**Figure 4:**
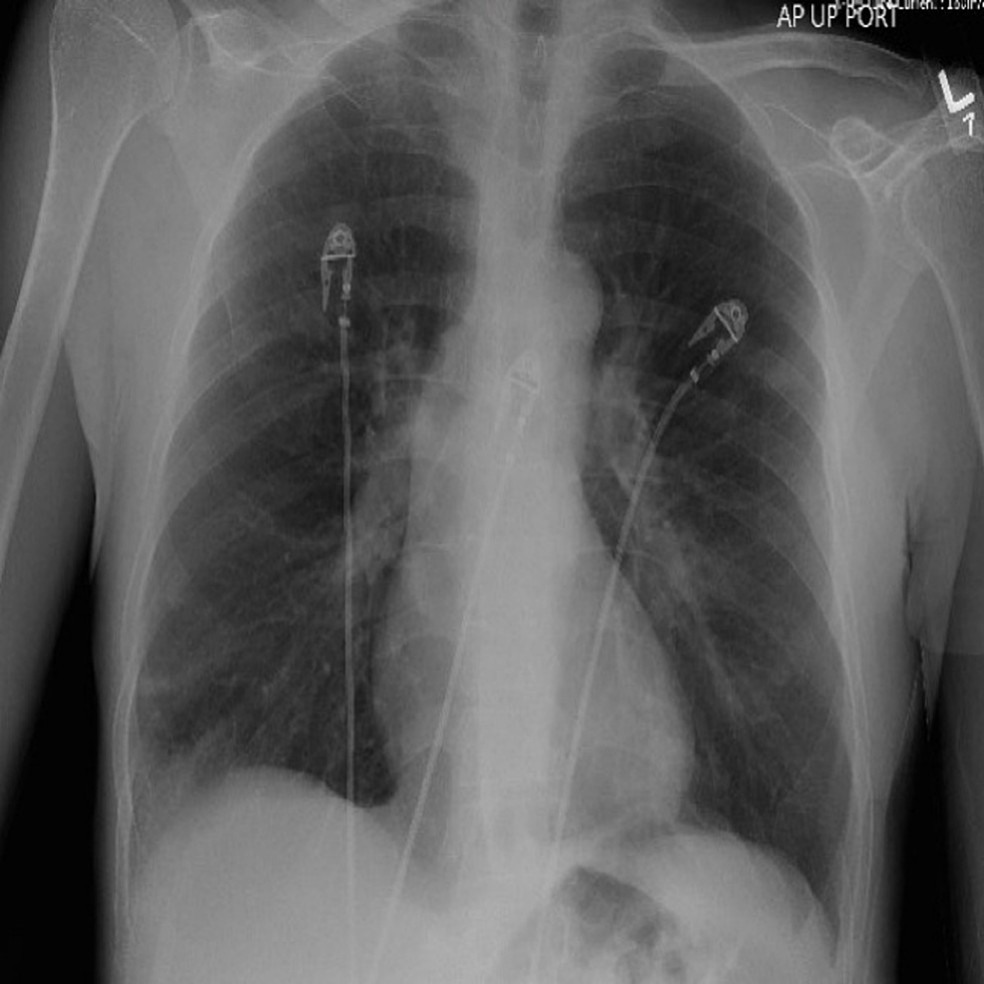
Bilateral nodular opacities on chest radiography

Circulating eosinophils in peripheral laboratory studies were elevated at 2.1 x10^3^/uL (24%) with a noted immunoglobulin E (IgE) of 1773 (normal <100). Human immunodeficiency virus (HIV) and stool parasites were negative. Chest radiograph performed in the emergency department incidentally revealed nodular opacities bilaterally (Figure 4).

**Figure 5:**
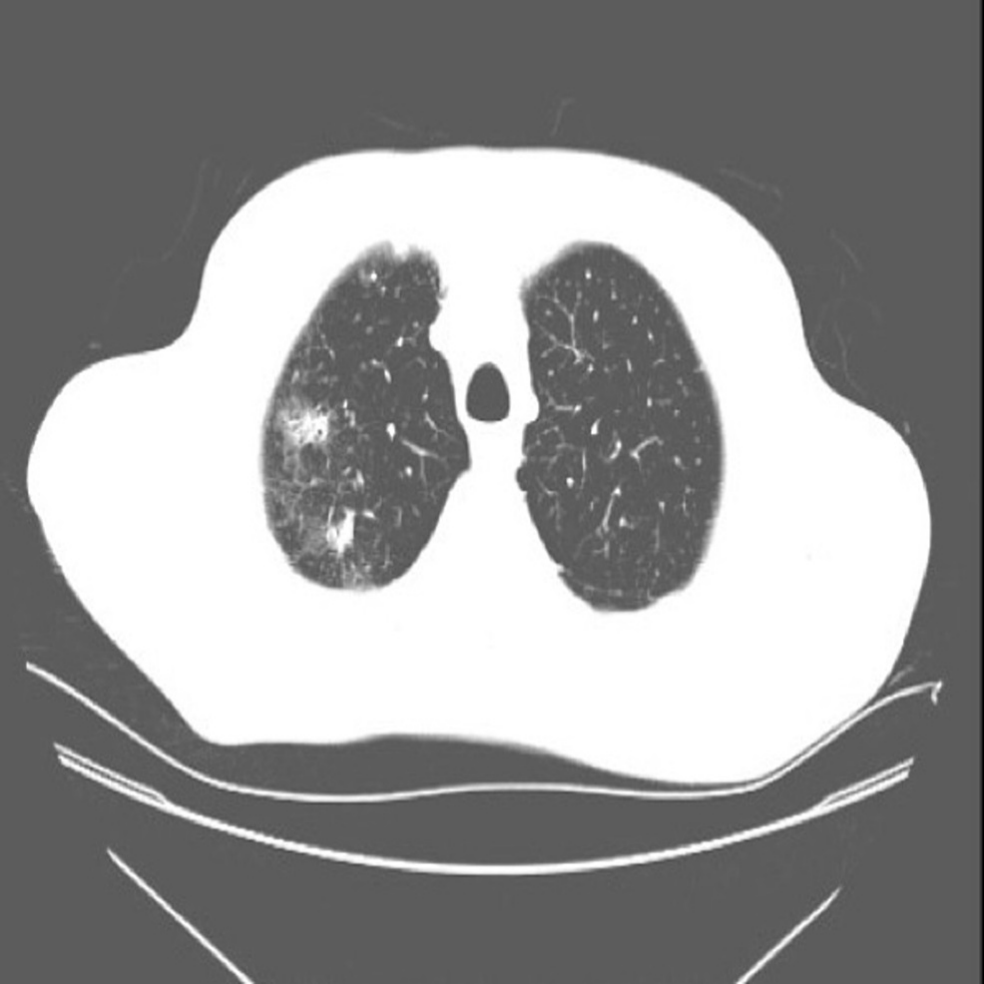
Reticulonodular infiltrates in the right upper lobe on CT

**Figure 6:**
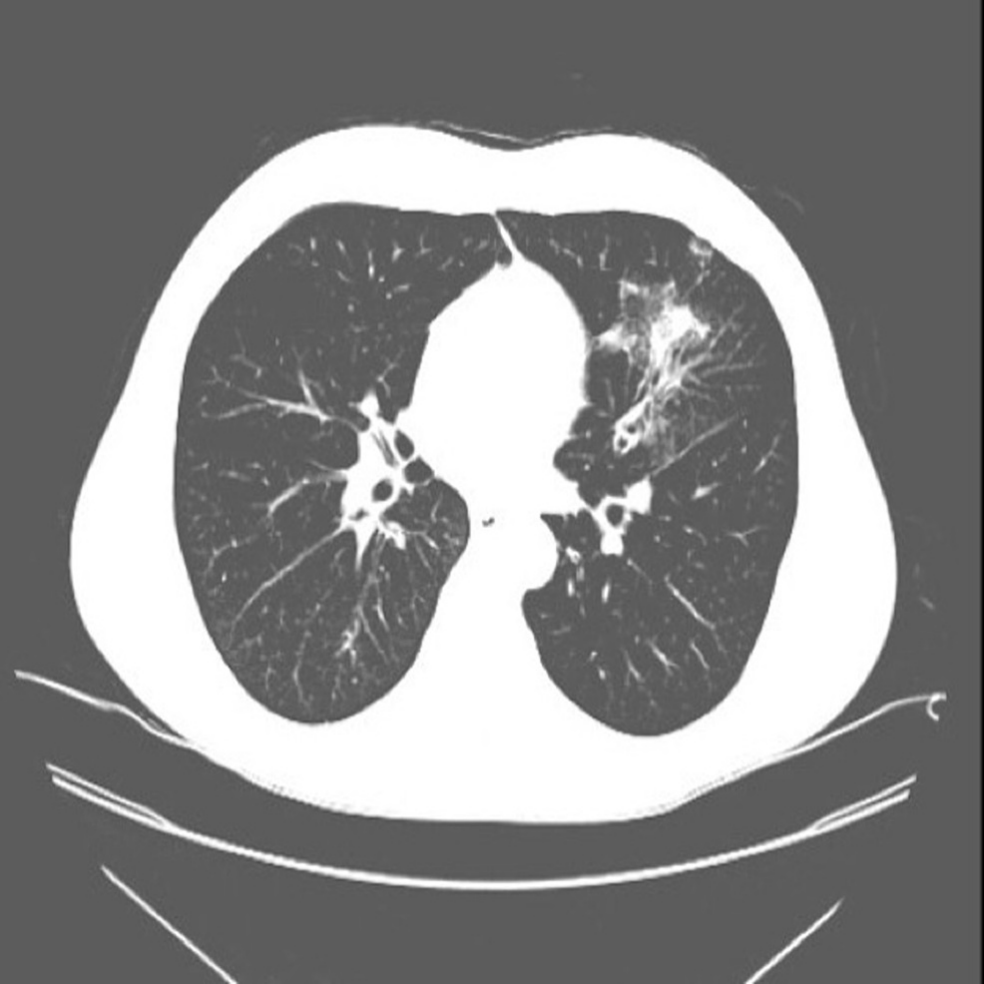
Reticulonodular infiltrates on the left lower lobe on CT

A follow-up CT scan showed multiple reticulonodular infiltrates (Figures 5-6).

The diagnosis of Löeffler’s syndrome was made secondary to cutaneous larva migrans given the patient’s presentation, laboratory results and findings on radiographic imaging. 

The patient was then administered a single dose of oral albendazole 400 mg. His dermatological lesions and severe pruritus improved. The patient fortunately continued to not have any respiratory complaints with oxygen saturations remaining stable on room air. He was ultimately discharged home with a course of oral doxycycline due to concerns of superimposed cellulitis with instructions to followup with an outpatient facility for routine surveillance of his serum eosinophilia and pulmonary infiltrates.

## Discussion

Cutaneous larva migrans is a condition secondary to the migration of parasites with one of the most common being *Ancylostoma braziliense *[4] in the southeastern United States. The hookworm lives in the gastrointestinal systems of both felines and canines with the parasitic eggs frequently found in their feces. These organisms then go on to infect their host via dermal invasion. Barefoot individuals seem to be most at risk, as they account for up to 95% of cases [3]. Shortly after inoculation, serpentine tracks soon develop as the nematode burrows which then allows it to travel anywhere from a couple of millimeters to several centimeters per day [2,3]. Fortunately, the organism cannot complete its full life cycle in humans and usually dies within months of inoculation. 

The diagnosis is made clinically after a careful review of the patient’s history. Given the very distinct clinical features, other dermatological conditions can effectively be ruled out. Biopsy is rarely useful since the parasite itself is often located beyond the superficial lesions making it very difficult to isolate [4,6]. Stool samples are also not helpful until there is gastrointestinal involvement [1].

The disease itself is self-limiting but anti-helminthics are commonly administered due to the severe accompanying pruritus. Pharmacologic options include oral albendazole 400 mg or ivermectin 200 mcg/kg. Oral albendazole is well tolerated due to its overall low dosage and relatively fast anti-helminthic activity. Regression of lesions is typically seen in 24 to 48 hours [7]. Oral ivermectin is another well-tolerated therapy but upon literature review, it appears to be slightly less efficacious for specific cases of cutaneous larva migrans with associated folliculitis [6]. The duration of both therapies can be adjusted depending on the severity of the patient's symptoms. Other options are available but found to be not nearly as effective as the aforementioned ones. Success with cryotherapy is rather limited due to both the difficulty in localizing the organism and its inherent ability to survive low temperatures. Topical anti-helminthics are another option but local irritation can occur due to the need for repeat applications. Recurrence rates also tend to be higher, especially in cases with widespread lesions as opposed to localized ones [7]. Finally, antibiotics are typically warranted since overlying bacterial folliculitis are fairly commonplace in these infestations [3]. 

Löeffler’s syndrome is a type of pulmonary eosinophilia syndrome commonly caused by parasites. In this specific instance, it is thought that the pulmonary manifestations arise secondary to a type one hypersensitivity reaction as the larvae migrate to the lung parenchyma, leading to massive activation of eosinophils [1,3]. In its asymptomatic form, it is usually self-resolving requiring no treatment. Symptomatic patients may be given extended courses of both anti-helminthics and corticosteroids. Serial chest imaging and laboratory studies should be obtained to ensure resolution, although this can take anywhere from weeks to several months. 

## Conclusions

In summary, Löeffler’s syndrome should be considered in the differential diagnosis where patients present with migratory pulmonary infiltrates, peripheral eosinophilia, and skin findings. Diagnosis is typically made clinically due to the overall vague diagnostic criteria, so the patient's history and presenting symptoms are of utmost importance. Treatment is typically not needed but can be tailored to the individual depending on their presentation, and severity of both their dermatologic and pulmonary manifestations.
